# *aP2*-Cre-Mediated Inactivation of Estrogen Receptor Alpha Causes Hydrometra

**DOI:** 10.1371/journal.pone.0085581

**Published:** 2014-01-08

**Authors:** Per Antonson, Marko Matic, Neil Portwood, Raoul V. Kuiper, Galyna Bryzgalova, Hui Gao, Sara H. Windahl, Patricia Humire, Claes Ohlsson, Per-Olof Berggren, Jan-Åke Gustafsson, Karin Dahlman-Wright

**Affiliations:** 1 Department of Biosciences and Nutrition, Karolinska Institutet, Novum, Huddinge, Sweden; 2 The Rolf Luft Center for Diabetes and Endocrinology, Karolinska Institutet, Karolinska University Hospital L1, Stockholm, Sweden; 3 Karolinska Institute Phenotyping Core Facility, Department of Laboratory Medicine, Karolinska University Hospital, Huddinge, Sweden; 4 Centre for Bone and Arthritis Research, Institute of Medicine, Sahlgrenska Academy, University of Gothenburg, Gothenburg, Sweden; 5 Center for Nuclear Receptors and Cell Signaling, Department of Biology and Biochemistry, University of Houston, Houston, Texas, United States of America; University of Bari & Consorzio Mario Negri Sud, Italy

## Abstract

In this study we describe the reproductive phenotypes of a novel mouse model in which Cre-mediated deletion of ERα is regulated by the *aP2* (fatty acid binding protein 4) promoter. ERα-floxed mice were crossed with transgenic mice expressing Cre-recombinase under the control of the *aP2* promoter to generate *aP2*-Cre/ERα^flox/flox^ mice. As expected, ERα mRNA levels were reduced in adipose tissue, but in addition we also detected an 80% reduction of ERα levels in the hypothalamus of *aP2*-Cre/ERα^flox/flox^ mice. Phenotypic analysis revealed that *aP2*-Cre/ERα^flox/flox^ female mice were infertile. In line with this, *aP2*-Cre/ERα^flox/flox^ female mice did not cycle and presented 3.8-fold elevated estrogen levels. That elevated estrogen levels were associated with increased estrogen signaling was evidenced by increased mRNA levels of the estrogen-regulated genes lactoferrin and aquaporin 5 in the uterus. Furthermore, *aP2*-Cre/ERα^flox/flox^ female mice showed an accumulation of intra-uterine fluid, hydrometra, without overt indications for causative anatomical anomalies. However, the vagina and cervix displayed advanced keratosis with abnormal quantities of accumulating squamous epithelial cells suggesting functional obstruction by keratin plugs. Importantly, treatment of *aP2*-Cre/ERα^flox/flox^ mice with the aromatase inhibitor Letrozole caused regression of the hydrometra phenotype linking increased estrogen levels to the observed phenotype. We propose that in *aP2*-Cre/ERα^flox/flox^ mice, increased serum estrogen levels cause over-stimulation in the uterus and genital tracts resulting in hydrometra and vaginal obstruction.

## Introduction

Estrogen receptor alpha (ERα, NR3A1) and ERβ (NR3A2) are two nuclear receptors that mediate the physiological responses to estrogen [1]. They are ligand-activated transcription factors, encoded by the *Esr1* and *Esr2* genes, that bind to DNA and regulate transcription in response to their ligands [2]. ERα has important roles in both the regulation of male and female reproduction, and also in the control of metabolism [3]. During the estrous cycle, 17β-estradiol (E2) levels are regulated via feedback mechanisms involving the ovaries, hypothalamus and pituitary gland. At the mid-stage of the estrous cycle, E2 produced within the gonads exerts a stimulatory effect on gonadotropin-releasing hormone (GnRH) neurons in the hypothalamus. The resulting GnRH discharge stimulates the anterior pituitary gland to release luteinizing hormone (LH), which in turn triggers ovulation. At other stages of the estrous cycle, E2 exerts a negative feedback that results in the suppression of GnRH secretion, as reviewed in [4]. Estrogenic signals, via ERα, also control a number of functions in the uterus including early events like hyperemia and water imbibition, and later events such as epithelial cell proliferation and differentiation [5], [6]. Mice lacking ERα, ERα^−/−^ mice, are infertile and have atrophic uteri that do not respond to E2 [7], [8], [9], [10], [11].

In this study, floxed ERα mice were crossed with *aP2*-Cre transgenic mice to generate *aP2*-Cre/ERα^flox/flox^ mice. Consistent with the well-described expression of the *aP2* promoter in adipocytes, *aP2*-Cre/ERα^flox/flox^ mice display down-regulation of the ERα transcript in both white and brown adipose tissue (WAT and BAT). However, down-regulation of the ERα transcript is also pronounced in the hypothalamus. Phenotypically, female *aP2*-Cre/ERα^flox/flox^ mice are infertile and develop hydrometra (fluid-filled distended uteri). We propose that in *aP2*-Cre/ERα^flox/flox^ mice, increased serum E2 levels cause over-stimulation of the uterus and genital tracts resulting in hydrometra and vaginal obstruction.

## Materials and Methods

### Ethics Statement

The “Stockholms Södra Djuretiska Nämnd” ethics committees approved all animal experiments (approval numbers: S10/09, S11/09, S17-11, S53/12 and S64/12).

### Animals

ERα^flox/flox^ mice (*B6.129X1-Esr1^tm1Gust^*) [7] were bred with transgenic mice expressing the Cre enzyme under the control of the *aP2*/*Fabp4* promoter (*B6.Cg-Tg(Fabp4-Cre) 1Rev/J*) [12] to generate mice with ERα deletion in fat cells. The final breeding step was performed using male *aP2*-Cre/ERα^flox/flox^ and female ERα^flox/flox^ mice. All mice analyzed in this study were on a congenic C57BL/6J genetic background. Genotyping of the ERα floxed locus was performed using PCR on DNA from ear or tail biopsies as described previously [7]. The presence of the *aP2*-Cre transgene was detected with primers 5′-GTTTCACTATCCAGGTTACGG and 5′- GTACTCTAAGTCCAGTGATC. Mice were maintained on a 14 h light, 10 h dark cycle and given a continuous supply of food and water.

### Fertility Tests

Fertility tests of female *aP2*-Cre/ERα^flox/flox^ (n = 6) mice were performed using continuous mating with male partners for three months. Mating was started at six weeks of age, and the numbers of litters and litter size were recorded.

### Estrous Cycle Stage Determination

Vaginal smears were collected from ERα^flox/flox^ (n = 2) and *aP2*-Cre/ERα^flox/flox^ (n = 5) mice using 0.9% saline as described elsewhere [13], placed on glass microscopy slides and viewed at 10×magnification.

### Measurement of Serum E2 Levels

E2 levels were determined using commercially available RIA kits (Siemens Medical Solutions, CA, USA), according to the manufacturer’s instructions.

### RNA Isolation and RT-PCR

Tissues were dissected from 6–9 week-old female mice and immediately frozen on dry ice for storage at −70°C. Total RNA was isolated from frozen tissues using Trizol reagent (Invitrogen) and then purified with RNeasy Plus Mini Kits (Qiagen) as described in [14]. cDNA was synthesized using random primers and either Superscript II (Invitrogen) or TaqMan® Reverse Transcription Reagents (Life Technologies). PCR was performed using RedTaq DNA polymerase (Sigma-Aldrich) and the following primers: ERα exon2F: 5′-CCCTACTACCTGGAGAACGA and ERα exon5R: 5′-TGCCCACTTCGTAACACTTG [7]. ERα expression levels were assessed by semi-quantitative real-time PCR with a 7300 Real-Time PCR System (Applied Biosystems), using TaqMan® Universal PCR Master Mix (Life Technologies) and a TaqMan® Gene Expression Assay (Mm00433148_mH, Life Technologies), according to the manufacturer’s instructions. Gene expression was normalized to 18S (TaqMan® Ribosomal RNA Control Reagents, Life Technologies). The expression of ERα target genes and Cre were analyzed using Power SYBR Green Master Mix (Applied Biosystems), according to the manufacturer’s instructions, and the following primers: Lactoferrin F 5′-CAGCAGGATGTGATAGCCACAA, R 5′-CACTGATCACACTTGCGCTTCT [15], Aqp5 F 5′-TTGTGAAGGCAGTGCAAGCT, R 5′-CACCCCTTTCTGGGATGGT [16]. Cre expression was analyzed with: Cre F 5′-GCCGCGCGAGATATGG and Cre R 5′-AGCTTGCATGATCTGCGGTATT.

### Histology

The female genital tract including ovaries, along with liver, kidney, brain, interscapular BAT, visceral (abdominal attached to ovaries and uterus) WAT, and inguinal subcutaneous WAT were collected from 12 mice that were between 6 and 12 weeks old. The tissues were fixed for 24 h in 4% neutral-buffered formaldehyde and stored in 70% ethanol prior to routine processing and embedding in paraffin blocks. Paraffin-embedded tissues were cut to 4 µm thickness, deparaffinized, rehydrated and stained with hematoxylin and eosin (H&E). The resulting slides were microscopically analyzed by a pathologist.

### Treatment with Aromatase Inhibitor

Letrozole was purchased from Selleckchem and dissolved at 2 mg/ml in saline containing 0.3% hydroxymethyl cellulose. Letrozole (10 mg/kg body weight) or vehicle control was delivered to mice via daily sc injection.

## Results

### Female *aP2*-Cre/ERα Knockout Mice are Infertile and have Increased E2 Serum Levels

**Figure 1:**
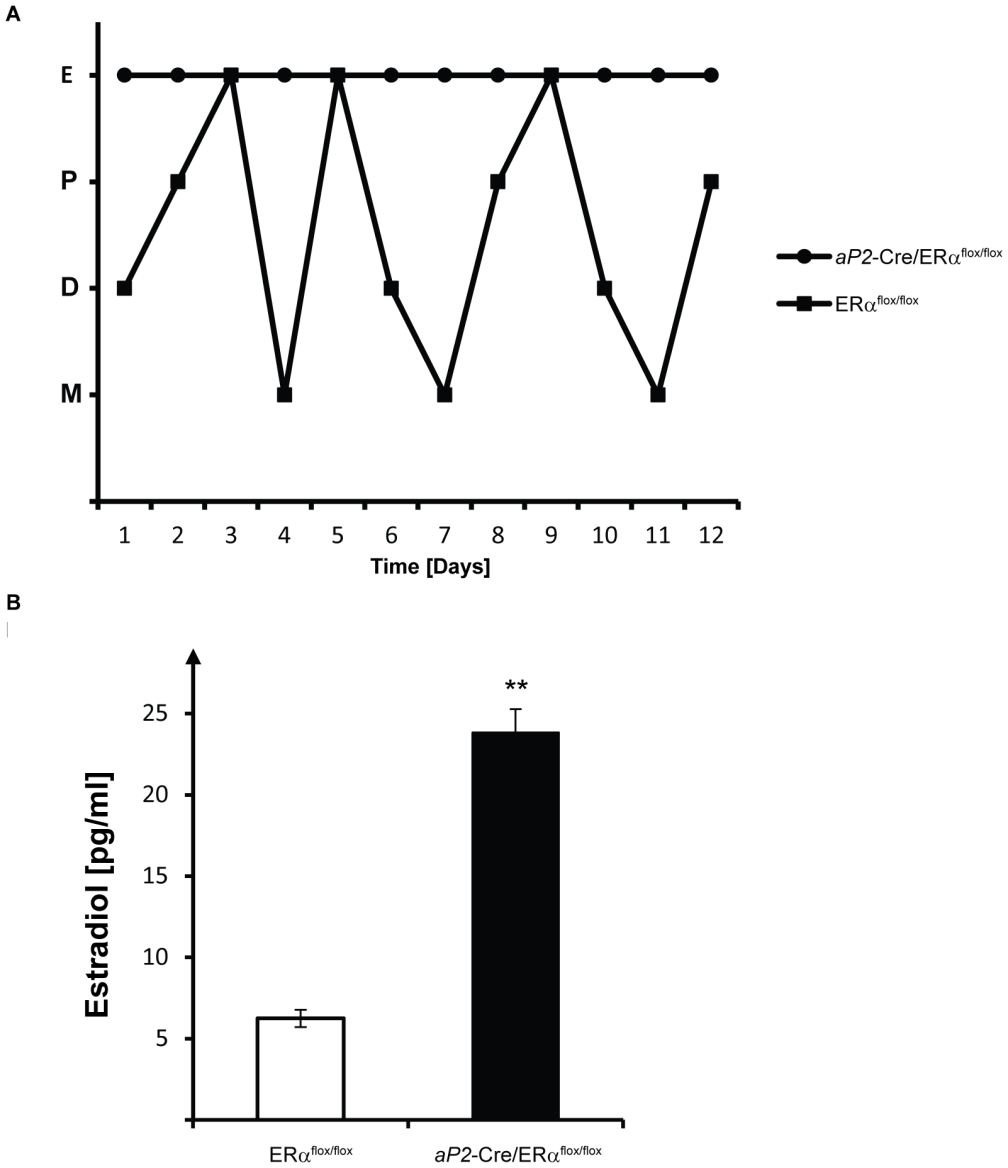
Lack of estrous cycle in *aP2*-Cre/ERα^flox/flox^ female mice. Vaginal smear analysis was performed on two month-old ERα^flox/flox^ and *aP2*-Cre/ERα^flox/flox^ mice on a daily basis for 12 days. (A) ERα^flox/flox^ females cycled, whereas *aP2*-Cre/ERα^flox/flox^ mice were in constant estrus, as determined by smears which consisted predominantly of cornified squamous epithelial cells. All graphs are representative and show two individuals out of totally seven analyzed. E, estrus; P, proestrus; M, metestrus; D, diestrus. (B) Serum E2 levels in ERα^flox/flox^ (n = 6) and *aP2*-Cre/ERα^flox/flox^ (n = 10) mice with ages between 10 and 14 weeks. Significance was determined by *t* tests. Error bars represent SEM, ***P<0.001.

Fertility in female *aP2*-Cre/ERα^flox/flox^ mice was investigated by continuous mating with fertile males for a three-month period. Since the breeding did not result in any pups (data not shown), we concluded that female *aP2*-Cre/ERα^flox/flox^ mice are infertile. Vaginal smears from 2 month-old *aP2*-Cre/ERα^flox/flox^ and ERα^flox/flox^ littermates demonstrated that while ERα^flox/flox^ animals cycled normally, *aP2*-Cre/ERα^flox/flox^ mice displayed vaginal smears compatible with constant estrus (Fig. 1A). We next analyzed serum E2 concentrations, and found elevated levels in *aP2*-Cre/ERα^flox/flox^ females compared to control female mice (Fig. 1B). The E2 levels were more than three-fold higher in *aP2*-Cre/ERα^flox/flox^ females compared to ERα^flox/flox^ female mice (23.8 pg/ml versus 6.25 pg/ml, respectively), and within the same range as our analysis of E2 levels in female mice with a global knockout of ERα (data not shown).

### ERα Expression in *aP2*-Cre/ERα Knockout Mice

**Figure 2:**
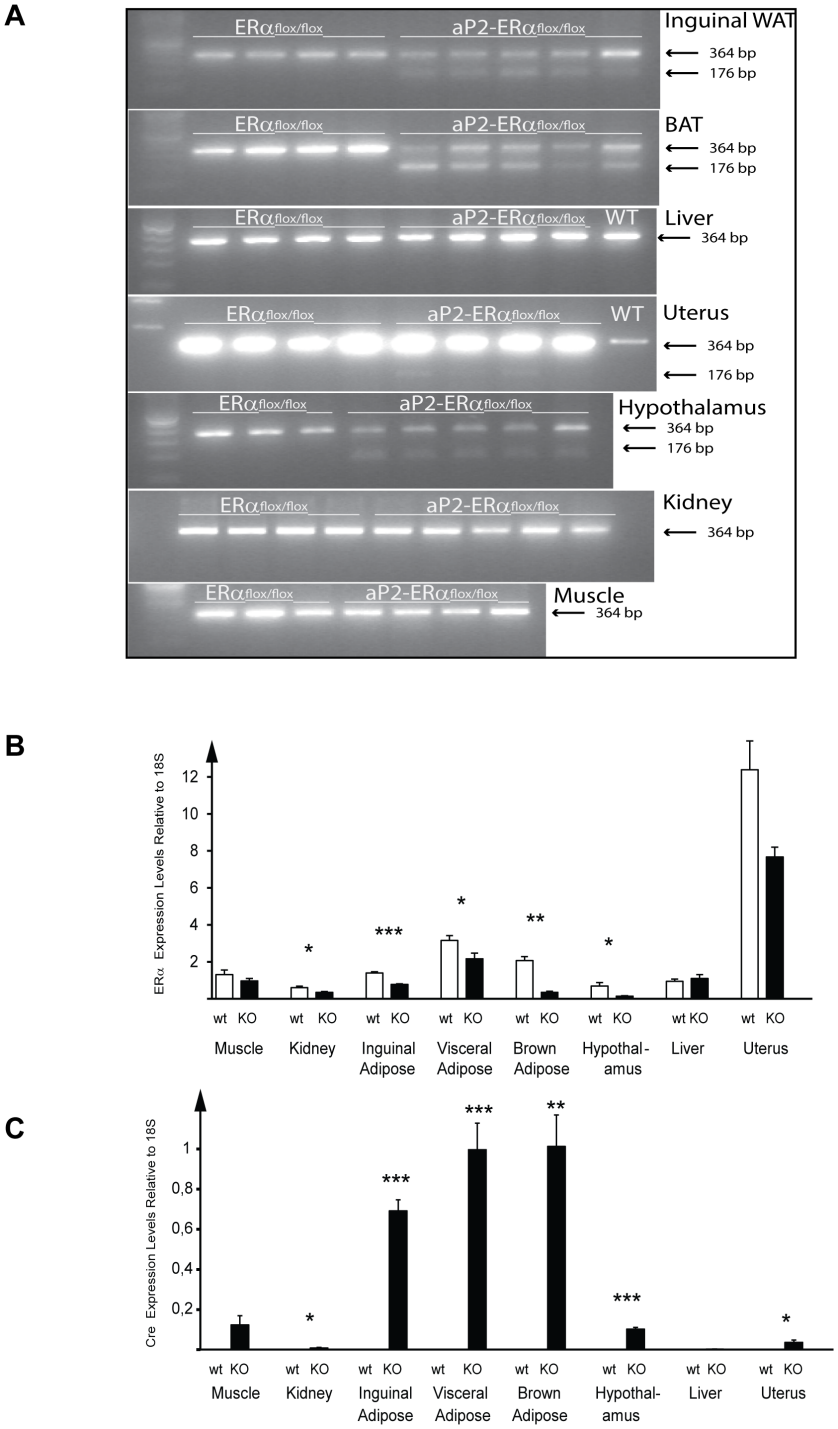
Specificity of ERα deletion in *aP2*-Cre/ERα females. (A) RT-PCR analysis of total RNA from inguinal adipose tissue, brown adipose tissue (BAT), liver, uterus, hypothalamus, kidney and muscle from ERα^flox/flox^ and *aP2*-Cre/ERα^flox/flox^ mice. Arrows indicate the WT ERα transcript (364 bp) and the Cre-deleted ERα transcript lacking exon 3 (176 bp). (B) Relative expression levels of ERα in muscle, kidney, inguinal adipose tissue, visceral adipose tissue, brown adipose tissue, hypothalamus, liver and uterus from ERα^flox/flox^ and *aP2*-Cre/ERα^flox/flox^ mice. (C) Relative expression levels of Cre in muscle, kidney, inguinal adipose tissue, visceral adipose tissue, brown adipose tissue, hypothalamus, liver and uterus from ERα^flox/flox^ and *aP2*-Cre/ERα^flox/flox^ mice. Values are given as mean ± SEM; *P<0.05, **P<0.01 and ***P<0.001 vs. control mice.

Ablation of the ERα gene in *aP2*-Cre/ERα^flox/flox^ mice was analyzed by RT-PCR utilizing primers flanking exon 3 of the ERα gene. This analysis generates a 364 bp product from the WT transcript and a 176 bp PCR product from the targeted allele that lacks exon 3. As expected, only the WT transcript was detected in samples from ERα^flox/flox^ mice. A 176 bp product, corresponding to the knockout transcript, was present in WAT and BAT from *aP2*-Cre/ERα^flox/flox^ mice (Fig. 2A). We also detected mRNA from the knockout allele in hypothalamus. Only the WT transcript was detected in liver and muscle. Traces of the knockout transcripts could also be detected in the uterus and kidney, although their levels are extremely low compared to those of the WT transcript in these tissues. To quantitatively determine the efficiency of knockout in the different tissues, mRNA levels of the WT ERα transcript were assayed using real time PCR. Significant down-regulation of the ERα transcript was observed in *aP2*-Cre/ERα^flox/flox^ mice, compared to control mice, in inguinal and visceral adipose tissue, brown adipose tissue, hypothalamus, and kidney (reductions of 44%, 31%, 83%, 80% and 42%, respectively), but not in muscle or liver; the reduction in ERα expression levels in the uterus was just below significance (Fig. 2B). To determine if the reduction of ERα mRNA levels in hypothalamus was due to Cre expression we assayed Cre expression using real time PCR. Significant Cre expression was detected in hypothalamus as well as all adipose depots analyzed and to lesser extents in uterus and kidney (Fig. 2C).

### Female *aP2*-Cre/ERα Knockout Mice Develop Hydrometra

**Figure 3:**
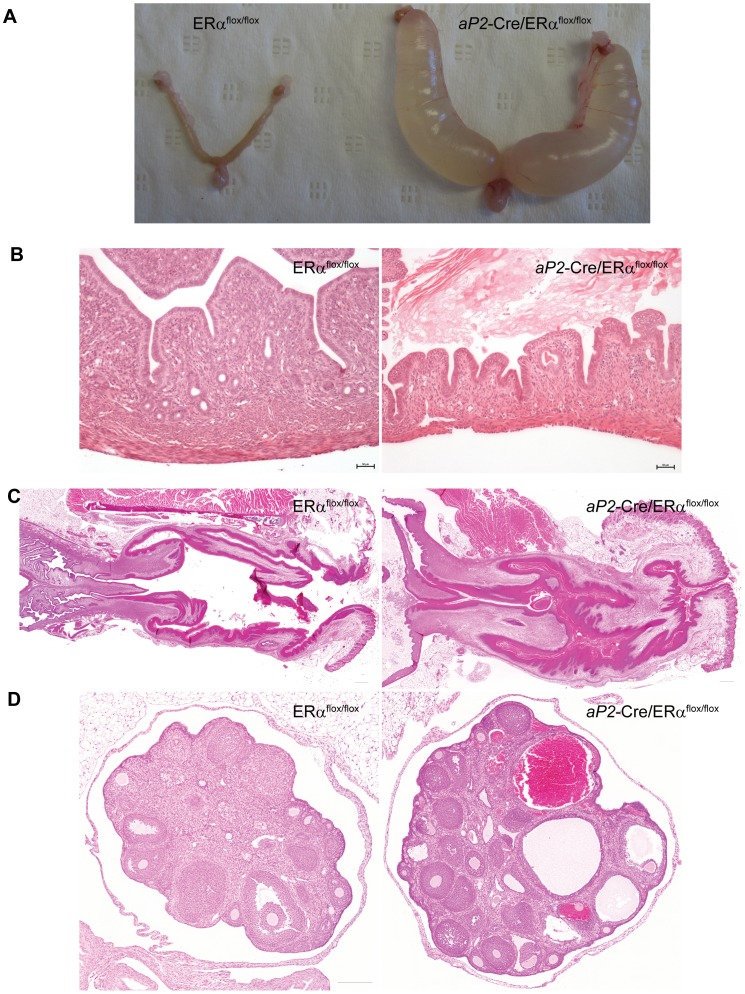
Defects in uterus and reproductive tract. (A) Representative images of uteri from five month-old ERα^flox/flox^ and *aP2*-Cre/ERα^flox/flox^ mice, showing that uteri from *aP2*-Cre/ERα^flox/flox^ mice are fluid-distended. (B) H&E staining was used to analyze morphological changes in the uteri. *aP2*-Cre/ERα^flox/flox^ mice have thin uterine walls with uterine distension together with atrophic muscles and glands. (C) Cervix and vagina from *aP2*-Cre/ERα^flox/flox^ mice have hyperkeratotic epithelium and vaginal debris consisting of accumulated cornified squamous epithelial cells. (D) Ovaries from 6 week-old *aP2*-Cre/ERα^flox/flox^ mice have hemorrhagic follicles and lack corpora lutea. Scale bar: 50 µm in (B) and 200 µm in (C) and (D).

All female *aP2*-Cre/ERα^flox/flox^ mice had swollen abdomens at 8 weeks of age. Internal anatomical examination revealed that the uteri in these mice were extensively fluid-filled (Fig. 3A). This phenotype was observed in all examined knockout mice but not in any of the control littermates. The accumulated uterine fluid was in most cases a clear and watery liquid characteristic of that seen in hydrometra, but in some of the animals the fluid was cloudy, consistent with an inflammatory response likely resulting from bacterial infection (pyometra).

Histologic analysis of the genital tract revealed that all investigated *aP2*-Cre/ERα^flox/flox^ mice had distended uteri, usually with watery contents (hydrometra) and thin walls, together with a vastly reduced glandular content and a thin muscular layer (atrophy) (Fig. 3B). All control mice showed normal uterus histology with well-developed muscular walls and glands.

Morphological analysis of the vagina and cervix (Fig. 3C) revealed that *aP2*-Cre/ERα^flox/flox^ mice had marked epithelial keratosis with abnormal quantities of accumulating cornified squamous epithelial cells in the vaginal lumen. We speculate that the observed hydrometra might have resulted from vaginal keratin plugs which could functionally obstruct the vagina. No other anatomical abnormalities, such as imperforate vagina or cervical/vaginal sagittal septa, were detected. Vaginal keratinization with variable luminal accumulation of cornified squamous epithelial cells was observed in 3 out of 6 normally cycling control mice.

The ovaries of *aP2*-Cre/ERα^flox/flox^ mice consistently demonstrated hemorrhagic follicles when compared to control mouse ovaries (Fig. 3D). Additionally, *aP2*-Cre/ERα^flox/flox^ ovaries did not show any signs of luteinisation, compared to control ovaries, which exhibited normal corpora lutea in 2 out of 3 observed specimens. Since antral follicles were abundant in all of the *aP2*-Cre/ERα^flox/flox^ ovaries examined, *aP2*-Cre/ERα^flox/flox^ ovaries appear to halt follicle development only at the final stage before ovulation. Indeed, the hemorrhages observed are likely the result of aberrant ovulation or follicle rupture. No marked differences in the numbers of atretic follicles were observed.

### Up-regulation of Estrogen Target Genes

**Figure 4:**
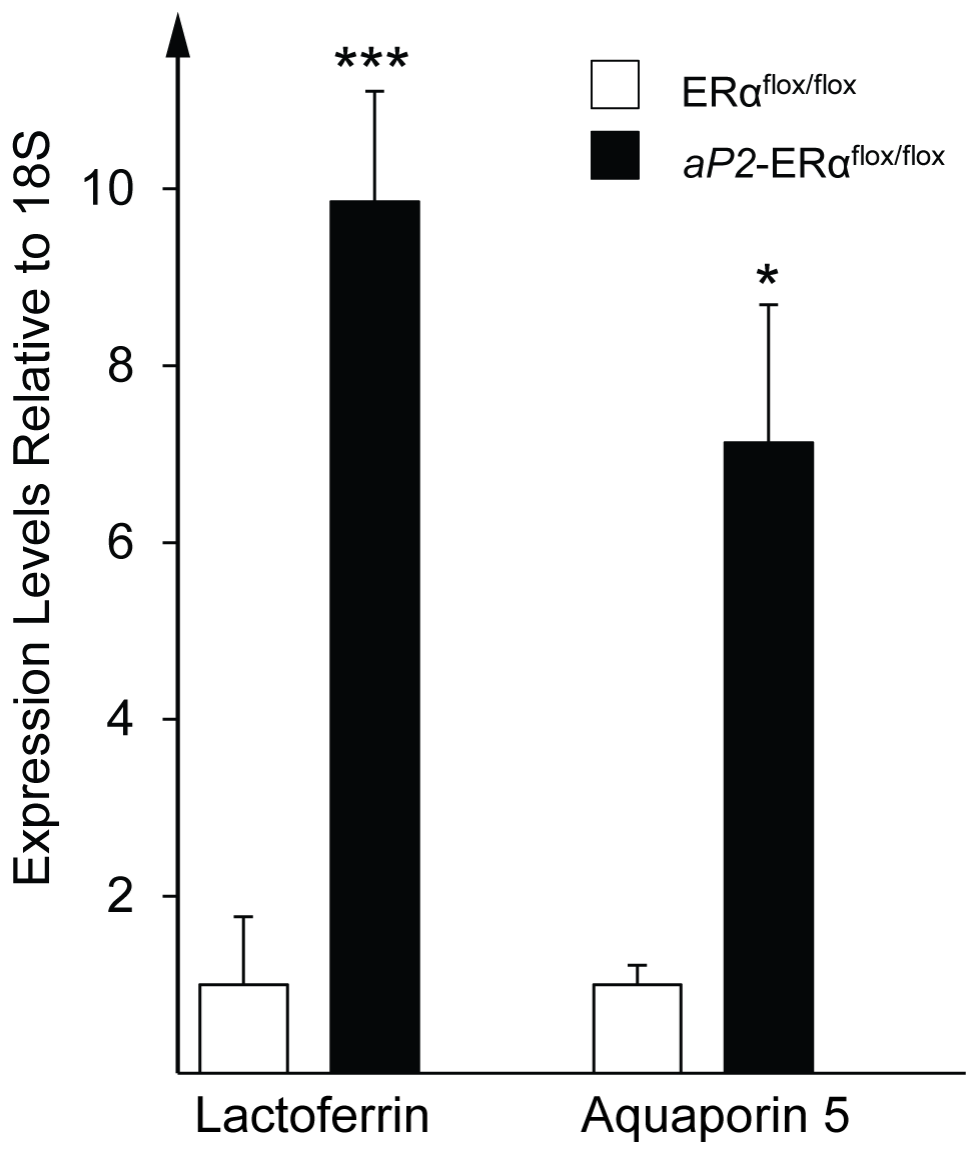
Quantitative PCR analysis of selected estrogen target genes. Relative expression levels of lactoferrin and aquaporin 5α^flox/flox^ and *aP2*-Cre/ERα^flox/flox^ mice. Values are given as mean ± SEM; *P<0.05 and***P<0.001 vs. control mice.

To identify genes in the uterus that could be involved in the development of hydrometra we analyzed the expression of the known E2 target genes, lactoferrin and aquaporin 5 [15], [16]. Lactoferrin mRNA levels were almost 10-fold higher, and aquaporin 5 mRNA levels about 7-fold higher, in the uterus of *aP2*-Cre/ERα^flox/flox^ mice compared to controls (Fig. 4).

### Inhibition of Endogenous Estrogen Synthesis Reduces Hydrometra

**Figure 5:**
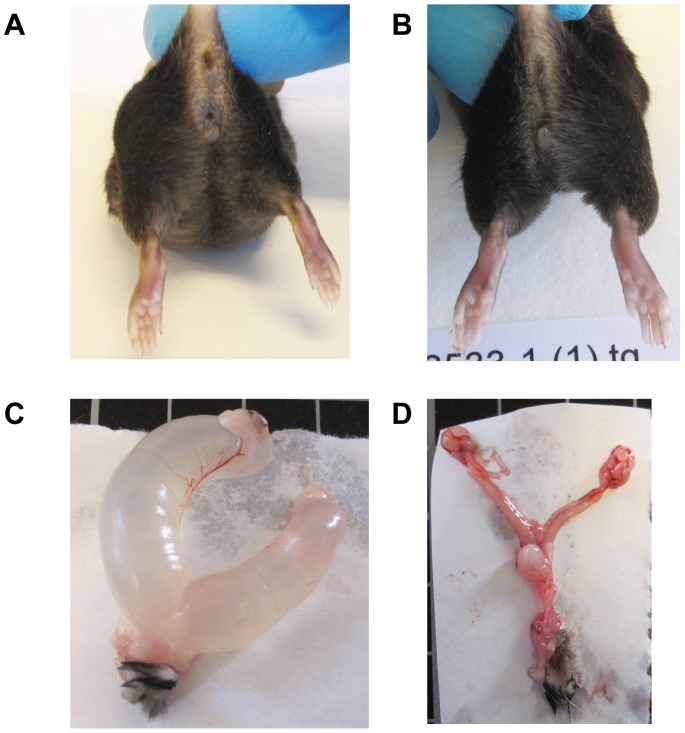
Uterus from mice treated with Letrozole. (A) 13 weeks old *aP2*-Cre/ERα^flox/flox^ mice treated with vehicle have swollen abdomen while littermates treated with Letrozole for 17 days looks normal (B). (C) Uterus from vehicle treated *aP2*−/ERα^flox/flox^ mice with severe hydrometra. (D) Uterus from Letrozole treated *aP2*-Cre/ERα^flox/flox^ mice looks normal.

To analyze if inhibition of endogenous estrogen synthesis via the aromatase enzyme would reverse hydrometra, we treated *aP2*-Cre/ERα^flox/flox^ (n = 5) and ERα^flox/flox^ (n = 5) mice with the aromatase inhibitor Letrozole or vehicle. Mice were 10 weeks old at the start of the treatment, at which stage all *aP2*-Cre/ERα^flox/flox^ mice displayed clear visual signs of hydrometra. Importantly, Letrozole treatment reversed visual signs of hydrometra in *aP2*-Cre/ERα^flox/flox^ (n = 3) mice within one week of treatment. When the mice were sacrificed after 17 days of treatment, uteri appeared grossly normal in Letrozole-treated mice while the vehicle-treated mice presented a severe hydrometra phenotype (Fig. 5A, B).

## Discussion

We generated a novel mouse line in which Cre-mediated deletion of ERα is regulated by the *aP2* promoter with the initial aim of targeting adipocyte ERα signaling using the Cre/loxP system. During the generation of this mouse strain, we observed that *aP2*-Cre-driven deletion of ERα leads to infertility in female mice and an arrest of the estrous cycle with hydrometra and increased serum E2 levels. Expression studies showed that the *aP2*-Cre transgene directs expression of Cre to adipose tissue as expected. Additionally, the *aP2*-driven Cre gene is also expressed in the hypothalamus, with a concomitant reduction in ERα levels also in this tissue (Fig. 2). The expression of Cre in the hypothalamus in *aP2*-Cre mice is consistent with previous observations [17], [18]. Estrogen action in the hypothalamus has drastic effects on the estrous cycle and on regulation of serum E2 levels [19], [20], [21], and it is conceivable that deletion of ERα in this brain region is the dominant cause of the severe reproductive effects observed in female *aP2*-Cre/ERα^flox/flox^ mice.

We speculate that the increased E2 levels observed in female *aP2*-Cre/ERα^flox/flox^ mice are related to deletion of ERα in the hypothalamus, resulting in disruption of the E2 feedback loop. Brain-specific ERα deletion using *CamKIIα*-Cre or *nestin*-Cre has previously been shown to cause elevated serum E2 levels and infertility [19], [21]. Also deletion of ERα in distinct hypothalamic neurons (*SF1*-Cre or *POMC*-Cre) affects fertility [21]. The increased levels of serum E2 in *aP2*-Cre/ERα^flox/flox^ mice may explain the block in the estrous cycle, since cycling E2 levels control the estrous cycle in mammals, exerting both negative and positive feedback effects [22], [23].

E2 is known to influence both uterine weight and vaginal epithelial cytology, and treatment of mice with E2 stimulates both uterine weight gain and vaginal epithelial proliferation and keratinization. In global ERα knockout mice, E2 treatment does not increase either uterine weight or the abundance of cornified epithelial cells in the vagina, showing that ERα is necessary for both these processes [8]. Long term treatment of WT mice with E2 has been shown to result in hydrometra [24], and we suggest that the hydrometra observed in *aP2*-Cre/ERα^flox/flox^ mice is a result of continuous E2 stimulation of the uterus, combined with severe vaginal hyperplasia and keratinization, resulting in accumulation of vast numbers of intraluminal keratinized squamous epithelial cells. In support of this, we show that treatment of *aP2*-Cre/ERα^flox/flox^ mice with the aromatase inhibitor Letrozole reverses hydrometra (Fig 5). Interestingly, short-term E2 treatment of global ERβ knockout mice also results in fluid-filled uteri, and it was speculated that this is a result of increased signaling by ERα due to the loss of ERβ which was suggested to dampen the effects of ERα [25]. A similar uterine phenotype was described by Wintermantel *et al*. [19] in mice with a *CamKIIα*-Cre-driven neuron-specific ERα knockout. In contrast, mice with a global deletion of ERα have severely hypoplastic uteri [7], [8], [9], [10], [11], although serum E2 levels are increased [8], [9], [26], [27], [28]. In this case, the uterus cannot respond to the increased E2 levels due to lack of ERα in this organ. Although ERα expression was also reduced in the uteri of *aP2*-Cre/ERα mice this reduction did not achieve significance, and was presumably insufficient to block overstimulation in this organ in response to elevated E2 levels, followed by atrophy at later stages. In line with this, we still observed marked up-regulation of the known E2 target genes lactoferrin and aquaporin 5 in uteri of *aP2*-Cre/ERα mice (Fig. 4).

In summary, we have generated a conditional ERα knockout mouse model using *aP2*-Cre–driven gene deletion, and we here demonstrate that these mice develop hydrometra. Our results are consistent with a mechanism involving reduction of ERα expression in the hypothalamus, which results in disruption of E2 regulation and increased serum E2 levels, leading to a block of the estrous cycle and hyper-stimulation of the uterus. Collectively, these results underscore the roles of E2 and ERα as main players in the development of hydrometra, and also the challenges associated with the use of the *aP2*-Cre transgene to target adipose gene expression.
